# Contact stresses, pressure and area in a fixed-bearing total ankle replacement: a finite element analysis

**DOI:** 10.1186/s12891-017-1848-y

**Published:** 2017-11-25

**Authors:** Nicolo Martinelli, Silvia Baretta, Jenny Pagano, Alberto Bianchi, Tomaso Villa, Gloria Casaroli, Fabio Galbusera

**Affiliations:** 1 IRCCS Istituto Ortopedico Galeazzi, Milan, Italy; 2 Laboratory of Biological Structure Mechanics (LaBS), Department of Chemistry, Materials and Chemical Engineering “Giulio Nattaˮ, Politecnico di Milano, 20133 Milan, Italy

**Keywords:** Contact stress, Ankle arthroplasty ankle osteoarthritis

## Abstract

**Background:**

Mobile-bearing ankle implants with good clinical results continued to increase the popularity of total ankle arthroplasty to address endstage ankle osteoarthritis preserving joint movement. Alternative solutions used fixed-bearing designs, which increase stability and reduce the risk of bearing dislocation, but with a theoretical increase of contact stresses leading to a higher polyethylene wear. The purpose of this study was to investigate the contact stresses, pressure and area in the polyethylene component of a new total ankle replacement with a fixed-bearing design, using 3D finite element analysis.

**Methods:**

A three-dimensional finite element model of the Zimmer Trabecular Metal Total Ankle was developed and assembled based on computed tomography images. Three different sizes of the polyethylene insert were modeled, and a finite element analysis was conducted to investigate the contact pressure, the von Mises stresses and the contact area of the polyethylene component during the stance phase of the gait cycle.

**Results:**

The peak value of pressure was found in the anterior region of the articulating surface, where it reached 19.8 MPa at 40% of the gait cycle. The average contact pressure during the stance phase was 6.9 MPa. The maximum von Mises stress of 14.1 MPa was reached at 40% of the gait cycle in the anterior section. In the central section, the maximum von Mises stress of 10.8 MPa was reached at 37% of the gait cycle, whereas in the posterior section the maximum stress of 5.4 MPa was reached at the end of the stance phase.

**Discussion:**

The new fixed-bearing total ankle replacement showed a safe mechanical behavior and many clinical advantages. However, advanced models to quantitatively estimate the wear are need.

**Conclusion:**

To the light of the clinical advantages, we conclude that the presented prosthesis is a good alternative to the other products present in the market.

## Background

Total Ankle Arthroplasty (TAA) has become a valuable procedure to relieve pain and restore functions in patients with osteoarthritis or rheumatoid arthritis. Newer implant designs with improved clinical results and longer term outcomes studies with satisfactory survival rates, increased the popularity of TAA to address end-stage ankle osteoarthritis preserving joint movement [15, 16]. In the past, high failure rates were associated to the first generation of implants due to loosening and subsidence at the bone-implant interface as a consequence of abnormal shear, compression and rotations [2–4, 8, 9, 11]. Loosening rates of the first generation two-component implants were found to be 60% and 90% after 5 and 10 years, respectively [11]. Further knowledge of the biomechanics associated with TAA, resulted in the second generation of implants that involved less bony resection and avoided cemented components with stem or peg fixation for primary stability. Second generation of TAAs was fixed-bearing, semi-constrained and two-component systems. These implants had the advantages to increase stability and reduce the risk of bearing dislocation. However, this second generation of implants led to increased polyethylene wear, symptomatic impingement and subluxation or dislocation of the components [11, 12]. The third generation of implants was less constrained, two- or three-component design. Although these implants offer majors advantages over the past designs which commonly failed due to loosening and osteolysis, the clinical outcomes are still less satisfactory than total hip and total knee arthroplasty [14]. A randomized prospective study conducted on 200 ankle replacements of the three-component Buechel-Pappas (BP) and the Scandinavian Total Ankle Replacement (STAR), found a six year survivor-ship of 86.5% [25].

The Zimmer Trabecular Metal Total Ankle (ZTMTA) replacement, available in the United States and in Europe, is the newest total ankle arthroplasty system. This implant belongs to the third generation of TAAs. The theoretical advantages, which are clinically unproven yet, are indeed attractive: the lateral transfibular surgical approach would enable surgeons to better visualize the anatomic center of rotation of the ankle, and due to the use of an assisted external fixator alignment system, bony resections are guided during the entire procedure, thus maintaining the integrity of the blood supply to the skin and potentially reducing the risk of wound complications [23]. Moreover, the lateral approach permits an extensive exposure to the ankle and to the subtalar joint in the surgical plane between angiosomes, thus maintaining the integrity of the blood supply to the skin and potentially reducing the risk of wound complications. However, due to the fixed-bearing design, there are some concerns related to polyethylene wear as possible consequences of high contact pressure [17].

Finite Element (FE) analysis is a consolidated method to investigate the mechanical behavior of prostheses and biological tissues because it has many advantages. First, it reveals all the mechanical parameters (e.g. stresses and strains) that are not always experimentally measurable; second, it allows performing parametric studies and doing structural analysis, in order to highlight areas of design weakness and suggest improvement possibilities; third, it allows performing quicker and cheaper analyses with respect to experimental testing. Although in vivo and in vitro conditions are not completely reproducible in finite element models, this method can give a clear view of the mechanical effect of a specific loading condition, and it can be used as a supporting tool by the industries and surgeons. In the last decades, some numerical studies have been performed to investigate the effect of the design features of each component, of the polyethylene thickness and of the ankle flexion angle [15]. It has been showed that, in a mobile-bearing implant, increasing the polyethylene thickness causes a more uniform distribution of the contact pressure, but increases the von Mises stresses at the edges [15]. In a semiconstrained prosthesis, Miller et al. [19] found the peak von Mises stress beneath the contact surface in two different configurations of the talar component. Jay et al. [13] compared the contact pressure, von Mises stresses and contact area in seven different implants, concluding that increasing the liner thickness and the articulating-surface area, the contact stresses may significantly decrease. A main problem in TAA studies is that a direct comparison is not always possible to do, due to the different investigated implants and the different boundaries and loading conditions. Indeed, many studies applied simple loading conditions and did not consider the action of the ligamentus apparatus. Reggiani et al. [21] investigated the effect of the gait cycle in a three component TAA including the ligaments, finding an uneven distribution of the contact pressure with small peaks in the demanding loading experience of gait. Thus, finite element analysis is a useful tool to compare the biomechanical behavior of a new prosthesis and to compare it with the other implants available on the market.

The objective of this study was therefore to investigate the contact pressure, the von Mises stresses and the contact area in the polyethylene component of the new ZTMTA with three different thicknesses, using a 3D finite element analysis.

## Methods

**Fig. 1:**
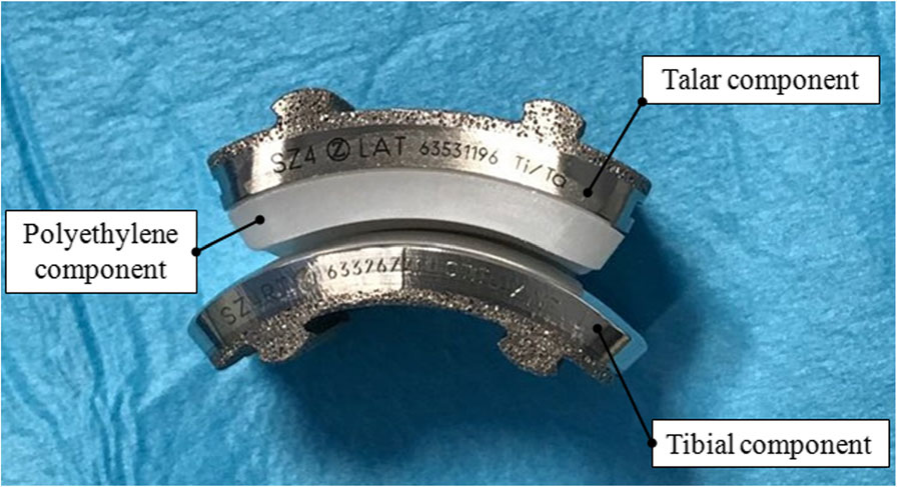
Zimmer Trabecular Metal Total Ankle Arthroplasty (TMTA). The implant is composed by the talar and the tibial component with a fixed-polyethylene bearing

A size two left ZTMTA and “zero” size of the polyethylene component (Fig. 1) provided by the manufacturer were digitally scanned (Scanprobe ST Nivol Scansystems, Pisa, Italy) and the initial Computer Aided Drafting (CAD) models were created (SolidWorks, Dassault Systemes S.A., Vlizy, France). The talar, the tibial and the polyethylene components were then smoothed and the imperfections due to the scanning process were removed.

**Fig. 2:**
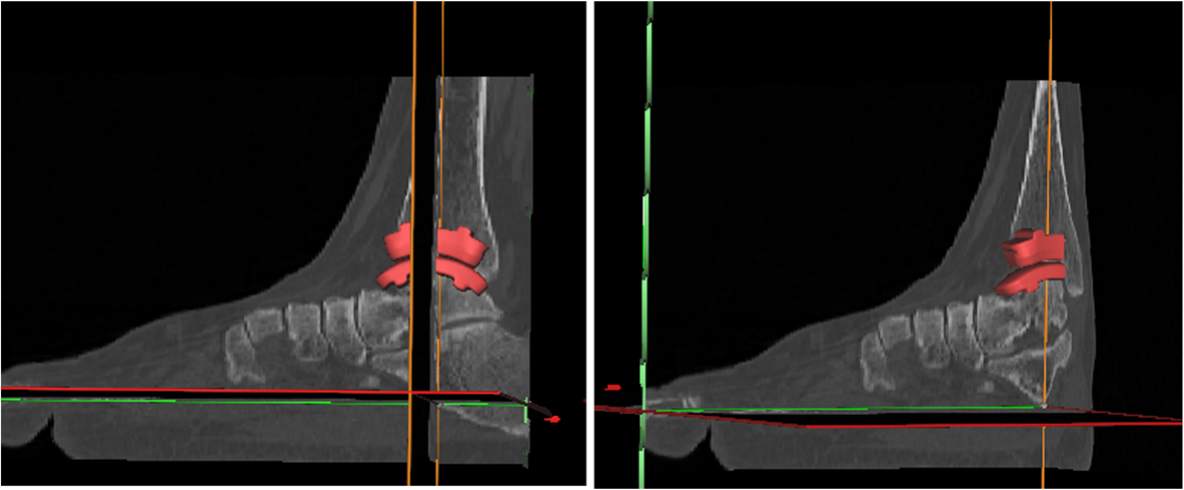
Schematic representation of the implant positioning using the CT images

Computed Tomography (CT) images, one in the sagittal and one in the frontal view, were acquired from a selected patient to assess the consolidation of the fibular osteotomy at the IRCCS Istituto Ortopedico Galeazzi (Milan, Italy). Written informed consent for the use of the data for research purposes was obtained and the patient’s data were anonymized. Because the patient was not involved in the study and the data were anonymized, ethics approval was not requested (CPMP/ICH/135/95, Good Clinical Practice GCP: Consolidated Guidance, European Directive 75/318/EEC). CT images were used to locate the ZTMTA model in neutral position using the software Mimics (Materialise, Leuven, Belgium) (Fig. 2). Then, 3D models were imported into ABAQUS (Version 6.12–1, Dassault Systemes) and meshed. The final assembled model was composed by 23,631 linear hexahedral elements.

**Fig. 3:**
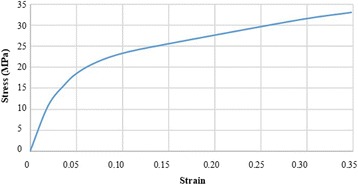
Stress-strain curve of the polyethylene material [16]

All the materials in the finite element model were homogenous, isotropic and linear elastic, except for the Ultra High Molecular Weight Polyethylene (UHMWPE) component that was modeled as an ideal elastic-plastic material [19] (Table 1). The stress-strain curve of the UHMWPE was taken from the literature and the yield stress was fixed to 10.86 MPa^19^ (Fig. 3). A surface to surface contact behavior was defined between the talar and the polyethylene components, with a friction coefficient of 0.04 [10]. Normal hard contact-overclosure behavior was assigned with the talar surface as master. To enforce the contact, a penalty method was applied. The tibial and the UHMWPE components were totally constrained and belong to the same mesh.

**Table 1:** Material properties assigned to the Zimmer Trabecular Metal Total Ankle components and to the bearing

Component	Material	Young Modulus (GPa)	Poisson ratio
Tibial	Ti-6Al-4 V	115	0.36
Talar	CoCrMo	241	0.3
Polyethylene	UHMWPE	Stress-strain curve taken from Miller et al. (2004) [17]

**Fig. 4:**
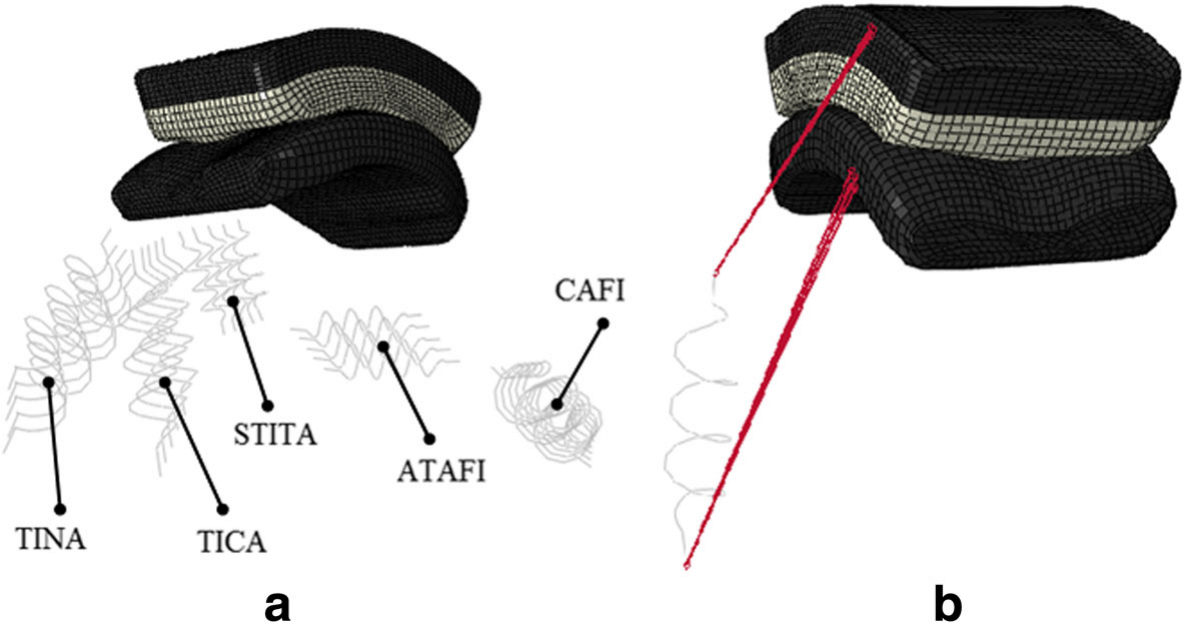
Schematic representation of the model with the ligaments (**a**) and an example of the ligament insertions (**b**). The tibial and the talar components are in dark gray, the polyethylene component is in light gray. Anterior Talofibular (ATAFI), Tibiocalcaneal (TICA), Calcaneofibular (CAFI), Tibionavicular (TINA) and Superior Tibiotalar (TITA)

The five major ligaments which surround the ankle joint complex were included into the model: the Anterior Talofibular (ATaFi), the Tibiocalcaneal (TiCa), the Calcaneofibular (CaFi), the Tibionavicular (TiNa) and the Superior Tibiotalar (sTiTa) (Fig. 4). The ligaments were modeled using non-linear, one dimensional spring elements, and their mechanical behavior was described by the constitutive law suggested by Funk and colleagues [7]. The ligaments were assumed having an elastic response described by the formulation

$$ \mathrm{F}\left(\upvarepsilon \right)=\mathrm{A}\left({\mathrm{e}}^{\mathrm{B}\upvarepsilon}-1\right) $$

where ε was the strain and A and B were taken from the literature (Table 2) [7].

**Table 2:** Elastic response function data of the ligaments (Funk et al.)

Ligaments	A(N)	B
ATaFi	7.18	12.50
CaFi	0.20	49.63
TiCa	0.51	45.99
TiNa	0.51	45.99
sTiTa	2.06	20.11

The locations of the ligaments insertions in the ankle neutral position were taken from previous studies [5, 21]. Since this model did not include any bones, a kinematic constraint was established between each insertion of ligament and one of the two components of the prosthesis. The origin of each ligament was linked to groups of nodes on the tibial component through a continuum-distributing coupling, whereas each insertion was constrained to a node set on the talus component (Fig. 4). According to Wei et al., in order to simulate the pre-tension of the ankle ligaments ε was set to zero for a spring element 2% shorter than the distance between the insertion points [24].

**Fig. 5:**
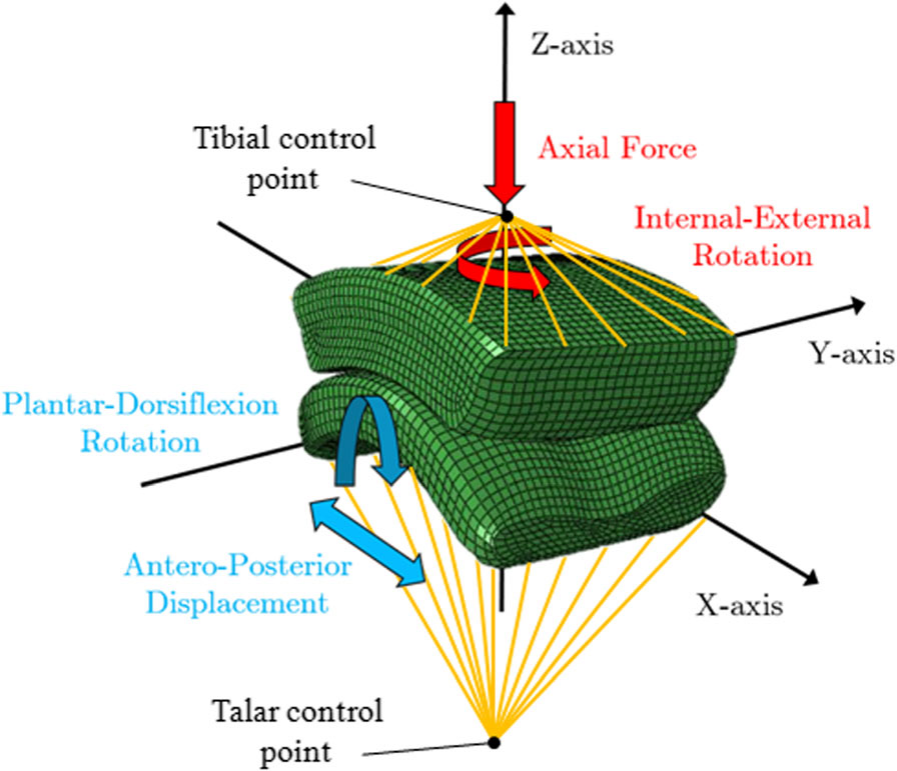
Schematic representation of the applied loading scenario (adapted from Bell et al., 2007). The axial load, the rotations and the displacement were applied to the tibial and to the talar control points, which were kinematically constrained to the relative components of the prosthesis

**Fig. 6:**
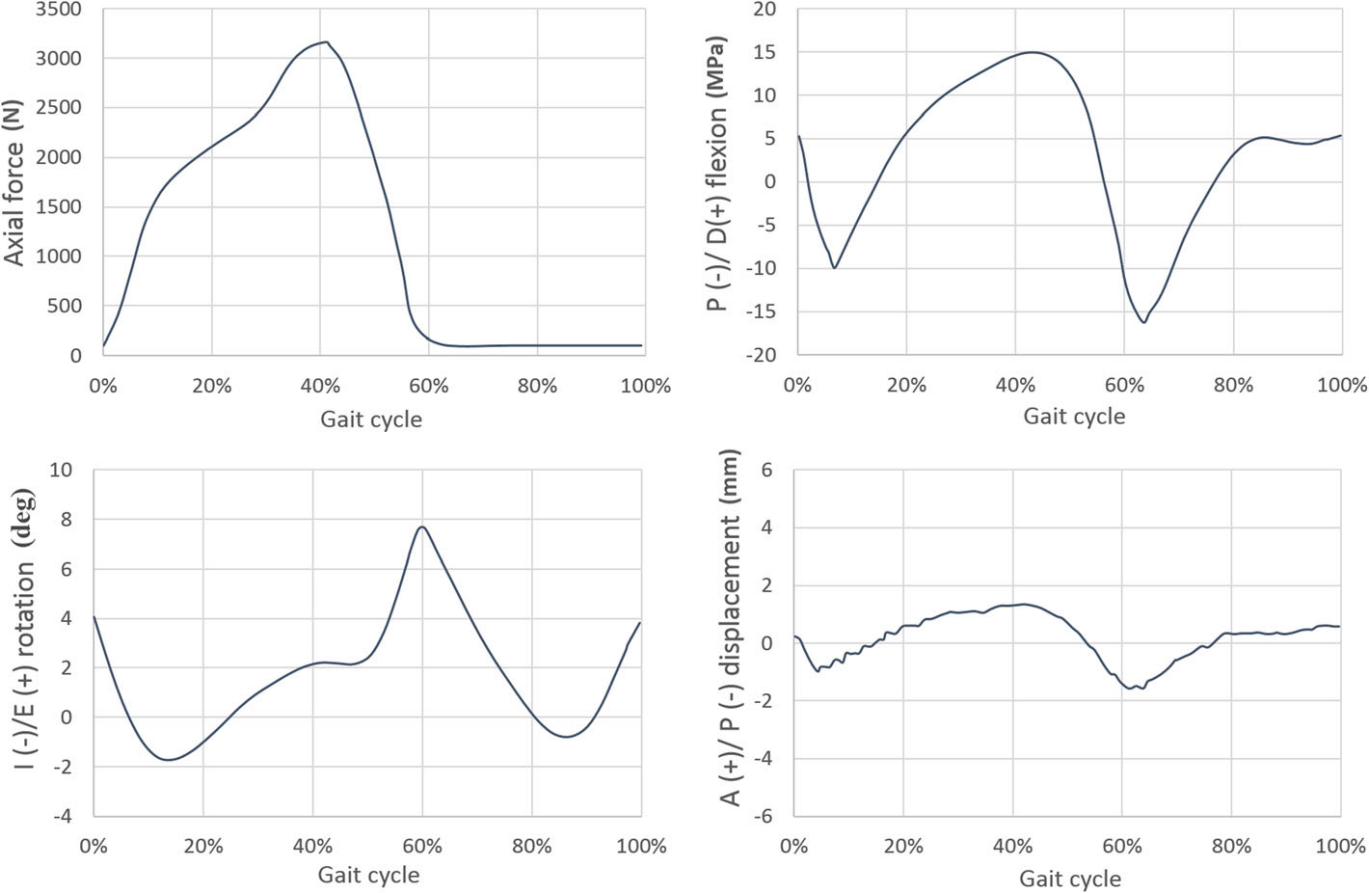
Time-histories of the applied boundary conditions during the gait cycle. A/P means Anterior/Posterior, I/E means internal/external, P/D means plantar/dorsiflexion. Adapted from Bell et al. (2007)

In order to apply rotations to the components of the prosthesis, a tibial and a talar control points were defined and kinematically constrained to the relative parts. The tibial component was constrained in the anteroposterior and mediolateral direction, whereas the talar component was constrained in the axial and in the mediolateral directions. The loading scenario proposed by Bell and colleagues [2] was applied: it included the axial compression and the internalexternal rotation of the tibial component, and the plantar dorsiflexion rotation and anteroposterior displacement of the talus (Fig. 5). The mechanical actions of muscles and tendons were included in the applied loads [1]. Each load was described by an independent time history (Fig. 6) [2]. Only the stance phase of the gait was investigated, which represented the 60% of the entire gait cycle. To ensure that the results of the simulations were independent on the element size, a mesh sensitivity study has been carried out. The maximum contact area between the talar and the polyethylene components, the maximum von Mises stress of the bearing and the peak contact pressure were evaluated for five element sizes equal in the entire assembly (1.35 mm; 1.3 mm; 1.0 mm; 0.7 mm; 0.6 mm). Because the value of the peak contact area converged to less than 0.3%, the von Mises stress converged to less than 0.2% and the maximum contact pressure converged to 1% difference upon the further mesh, the mesh with 1 mm element size was used.

The influence of polyethylene height was evaluated by analyzing two additional configurations, one 2 mm and one 4 mm thicker than the standard size. Thus, two additional models were created extruding the surface of the polyethylene component. The new assembled models were composed by 26,621 and 29,676 linear hexahedral elements, respectively. All the other parameters, as well the ligaments initial strain, were left unchanged.

For a more precise prediction of the stress distribution, the anterior, central and posterior cross sectional areas of the polyethylene component were investigated.

## Results

**Fig. 7:**
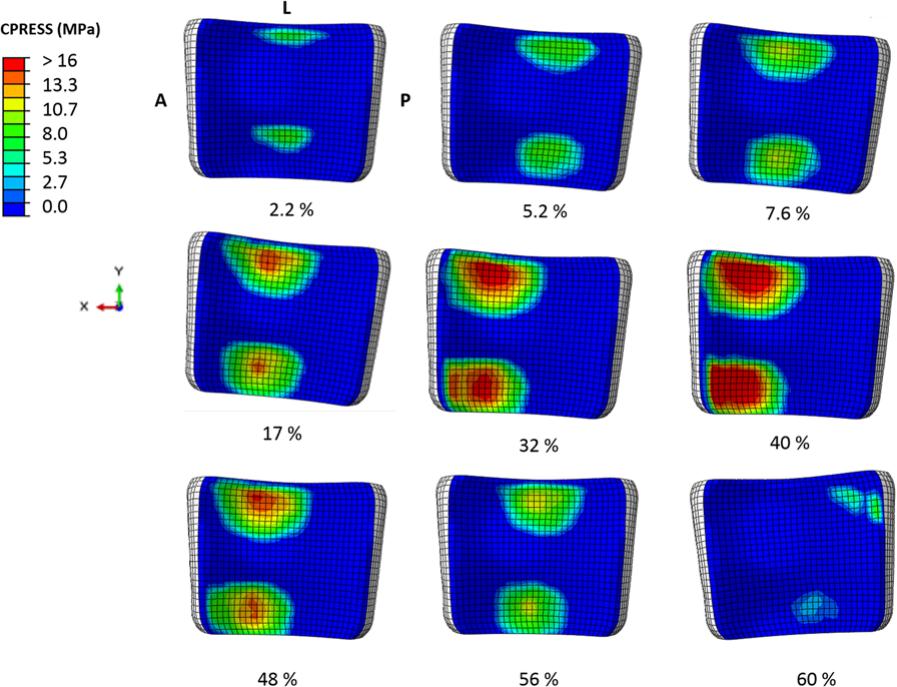
Contact stresses on the polyethylene bearing at different percentage of gait cycle. A = anterior, P = posterior, L = lateral

**Fig. 8:**
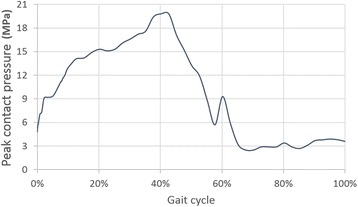
Peak contact pressure on the articulating surface during the entire gait cycle

The average and the maximum von Mises stress and contact pressure value were found between the 30–50% of the entire gait cycle in the anterior and in the central section (Table 3). The contact pressure distribution was higher in the anterior part during the most of the stance phase (Fig. 7). The peak values were found in the anterior region of the articulating surface, where reached 19.8 MPa at 40% of the gait cycle (Fig. 8). The average contact pressure during the stance phase of gait was 6.9 MPa.

**Table 3:** Average and maximum von Mises stress and contact pressure in the anterior, central and posterior section of the polyethylene component between 10 and 70% of the gait cycle

	Anterior				Central				Posterior			
	von Mises (MPa)		Contact pressure (MPa)		von Mises (MPa)		Contact pressure (MPa)		von Mises (MPa)		Contact pressure (MPa)	
	Average	Max	Average	Max	Average	Max	Average	Max	Average	Max	Average	Max
10%	1.3	4.9	1.2	7.6	4.2	9.4	5.7	12.4	0.8	2.3	0.0	0.0
20%	4.1	10.3	5.6	12.6	3.7	8.6	5.0	11.7	0.5	1.6	0.0	1.0
30%	6.1	11.9	8.4	16.2	2.7	7.5	3.3	11.0	0.3	1.0	0.0	2.0
40%	7.5	14.1	10.0	19.8	2.7	7.6	2.9	11.0	0.3	1.0	0.0	3.0
50%	2.8	6.9	3.4	8.6	4.3	9.7	6.1	13.2	0.6	2.2	0.4	4.9
60%	0.3	2.1	0.0	0.0	0.4	2.1	0.1	0.9	0.6	5.4	0.0	0.0
70%	0.4	2.5	0.1	2.5	0.2	0.9	0.1	0.9	0.1	0.2	0.0	0.0

**Fig. 9:**
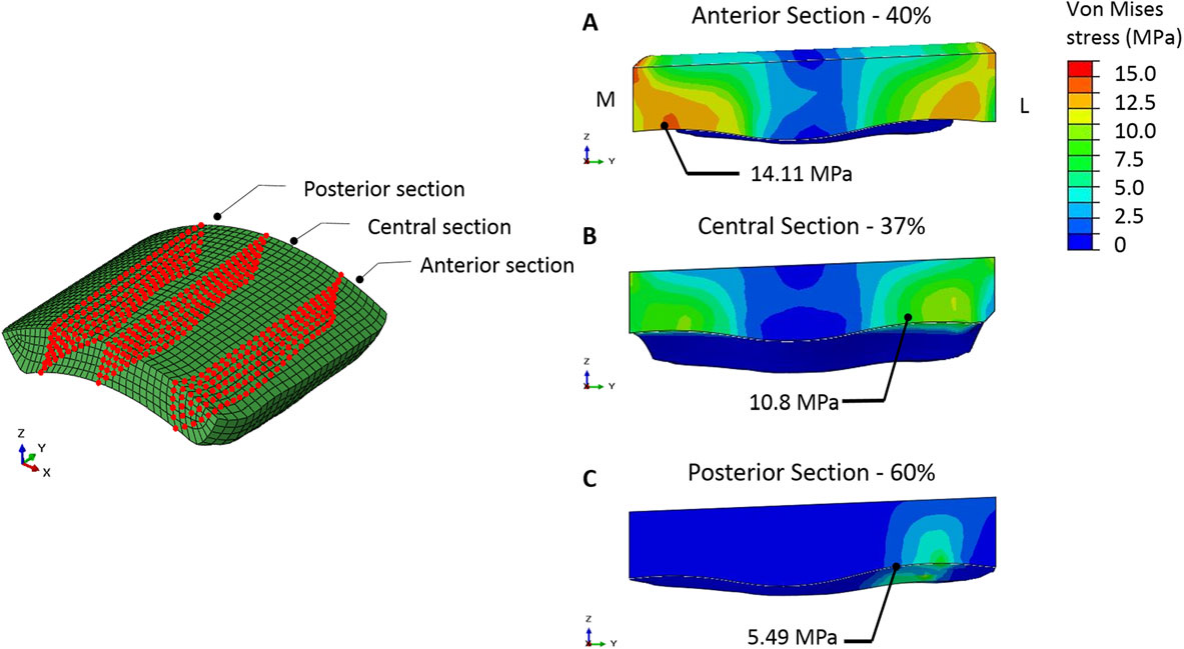
Sections for the investigation of stresses distribution on polyethylene component and maximum von Mises cross-sectional values for the anterior (A), central (B) and posterior (C) section, from the medial (M) to the lateral (L) side

**Fig. 10:**
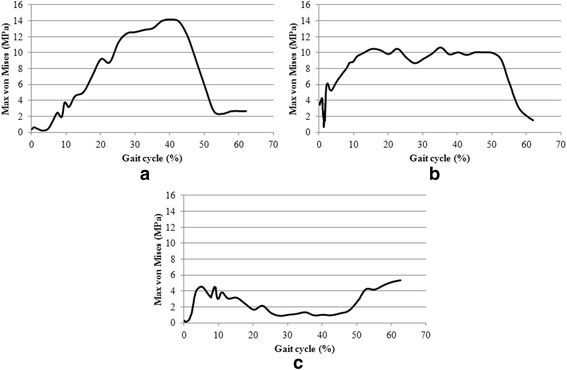
Maximum von Mises stress in the stance phase in anterior (**a**), central (**b**) and posterior section (**c**)

In the anterior section, the maximum von Mises stress of 14.1 MPa was reached at 40% of the gait cycle. In the central section, the maximum von Mises stress of 10.8 MPa was reached at 37% of the gait cycle, whereas in the posterior section the maximum of 5.4 MPa was reached at the end of the stance phase (60% of the gait cycle) (Figs. 9 and 10).

The contact area increased up to 296 mm^2^ (about 40% of the contact surface) until 35% of the gait cycle. This result was consistent with the maximum value of the set of loads and boundaries on the polyethylene bearing, in particular with the axial force. At the beginning of the stance phase, when heel strike occurred and the minimum solicitations acted on the polyethylene bearing, the minimum value of 28 mm^2^ (about the 4% of the contact surface) was reached.

The maximum lateral displacement and the maximum rotation of the polyethylene bearing were 1.67 mm and 1.4°, and were reached at about 40% and 62% of the gait cycle, respectively.

The talar component had a maximum internal rotation of 4.9° at about 18% of the gait cycle and a maximum external rotation of 3.8° at about 62% of the gait cycle.

Increasing the thickness of the polyethylene bearing did not significantly influence the von Mises stress and the pressure in the polyethylene bearing. The average and peak pressure values were 6.93, 6.32 and 6.78 MPa and 12.19, 11.50 and 12.33 MPa for the standard, 2 mm and 4 mm thicker polyethylene bearing, respectively. Von Mises stress was higher in the anterior section with respect to the lateral and the posterior ones in all cases, and was higher in the medial and in the lateral regions than in the central one. Increasing the thickness of the polyethylene component caused a reduction of the 2% and of the 9% the maximum von Mises stress in the anterior section in the 2 mm and 4 mm thicker bearing, respectively.

## Discussion

This study presents a numerical model of a novel TAR with three different sizes of fixed polyethylene bearing. Contact pressure, von Mises stresses and contact area were predicted during the stance phase of the gait cycle. The knowledge of these mechanical parameters is important because they are related to wear problems as well as to the risk of loosening and osteolysis.

In general, the model predictions were in a good agreement with previous studies [19, 22, 23] conducted on other total ankle replacements. However, the comparison of the present results with the literature was difficult due to the different designs analyzed, to the different loading protocols and boundary conditions and to the presence (or lack) of ligamentous apparatus.

For each studied configuration, it was showed that the average pressure value at the interfaces of ZTMTA was less than 10 MPa, which is generally considered a critical value for polyethylene integrity [9, 19]. However, peaks pressure higher than 10 MPa were recorded in small areas. High peaks pressure indicated local high stress concentrations of the polyethylene, which could cause wear, accelerated component loosening and clinical failure. Many contrast results were found in the literature. Some studies reported a yield stress values for medical grade polyethylene in the range from 15 to 23 MPa [19, 20]. Espinosa et al. [6] performed a finite element comparison between two validated models of the Agility two-component prosthesis (size 4 - DePuy) and the Mobility three-component prosthesis (size 3, 3 × 7 mm mobile-bearing - DePuy). They measured the contact pressure under standard position and under misalignments deviated from this relative position. The three-component Mobility TAA showed a contact pressure more evenly distributed and threefold lower than the two-component Agility, which exceeded the yield stress of 18 MPa for all tested configurations. Reggiani et al. showed that in the three-component Bologna-Oxford prosthesis [21] (BOX) the average contact pressure experienced by a lower surface of the polyethylene bearing was 10.3 MPa, with a peak value of 16.1 MPa at 79% of the stance phase (about 40% of the entire gait cycle). However, the applied loads and boundary conditions differed from this study: in particular, a lower axial load of 1600 N in combination with an anteriorposterior force on the talar component and an internal/external torque were applied. Saad et al. developed a FE model of the BOX implant and predicted a peak contact pressure of 18.4 MPa at 79% of the stance phase [21]. Differently from our study, they applied an internal/external rotation instead of a torque moment.

Von Mises stress is a mechanical condition used to indicate the state of stress in ductile materials and it is used to investigate the failure processes. In biomechanics, von Mises stress is a valuable indicator of the immediate post-operative period and for the implant failure. The von Mises stress values predicted in the current study were similar to the outcomes of previous FE models. Indeed, Miller et al., in an Agility FE model, found von Mises stress values in the range from 19.5 to 16.3 MPa in the edge and from 11.4 to 9.3 MPa in the center of the polyethylene bearing [19]. Jay et al. [13] analyzed the average von Mises cross-sectional stresses on the ankle joint subjected to an uniform pressure corresponding to the normal component of the ankle gait with a maximum value of 4400 N. The values reported ranged from 4 to 14 MPa; in our model, the average von Mises stresses on the polyethylene bearing ranged from 7 to 12 MPa.

Increasing the thickness of the polyethylene bearing showed a slight decrease of the von Mises stresses. This outcome was in agreement with the study of Bartel and collegues [1], which showed that in total knee replacement a polyethylene insert with a thickness of more than eight to ten millimeters should be maintained. This result suggested that the choice of the bearing size can be made on the basis of clinical reasons. On contrast, McIff and colleagues [18] found a noticeable effect of the polyethylene insert on pressure distribution: in particular they concluded that with a thicker polyethylene insert the pressure was more evenly distributed in the anteroposterior direction, while a thinner polyethylene resulted in higher stresses at the midline of the insert. The contact area between the talar and the polyethylene component has two conflicting mechanical aspects related: indeed, an extended contact area enhances the stability of the prosthesis, preventing possible dislocation of the implant. On the other hand, when the surface increases also the area subjected to wear could increase. It was showed that the contact surface increased with the load and it was always about the 25% of the natural ankle joint. This outcome ensured the stability of the prosthesis [1], but further studies should be done to investigate the wear generation. Jay et al. [13] conducted a FE investigation on seven Wright State University patented TAA models belonging to two different generations of implants. They found that neither coefficient of friction nor material properties differences of the talar and tibial component contributed to significantly change the stress state in the UHMWPE liner. They hypothesized, it was due to the fact that geometry had a major role in determining the stress state, concluding that increasing the liners thickness and the contact area could significantly decrease the cross-sectional von Mises stresses.

Some limitations related to the modeling approach should be highlighted. First, bones were not taken into account in the present model [9, 19]. However, this simplification was not expected to have a major impact on the prediction of the stresses on the contact surfaces because of the high Young modulus of the ZTMTA components (Table 1). Therefore, for mechanical reasons the load is supported by the implant, making the absence of bone a reasonable assumption. Moreover, a total osteointegration between the implant and the bone has been supposed, since any relative motions were neglected. Thus, we believe that this is a reasonable simplification, since the main purpose of the study was the evaluation of contact outputs on the polyethylene bearing components and not to investigate the state of stress in the bone. Caution may be taken to interpret the results because the model was not validated and an in vitro study may be performed to validate the current numerical findings. However, the ligaments properties were taken from the literature and the implants geometrical and mechanical parameters were given by the manufacturer; therefore we assumed the presented model reliable.

A major limitation of the present study regards the lack of a general consensus about the boundary and loading conditions to be used to simulate the gait cycle. Indeed, there are wide differences in the ankle loads used by various authors (e.g. among Bell and Reggiani), which may have a significant impact on the stress predictions [2, 21]. An additional limitation is that we analyzed only one size implant. In fact, the dimensions of the prosthesis may influence the pressure in the polyethylene component; however, it can be argued that larger implants are generally used for heavier patients, therefore a higher compressive force should be applied and the pressure distribution should result similar.

## Conclusions

In conclusion, taking into account these limitations of the modeling approach, the present results showed that the von Mises stresses and pressure values of the ZTMTA are generally similar to those calculated in previous studies for other implants, and a similar wear behavior in the long term might be expected. It should be noted that other factors not related to the biomechanical ones (e.g. invasivity, safety of the surgical access, etc.) should be taken into account when selecting a TAA and might provide advantages for the ZTMTA with respect to alternative implants. However, to the light of the clinical advantages, the minimum bone resection requirement and the similar mechanical outcomes between this study and others present in the literature, we conclude that the ZTMTA is a good alternative to the other prostheses present in the market.

Potential benefits need however to be proven by means of long term clinical studies.

## Abbreviations

ATaFi: Anterior Talofibular
BOX: Bologna-Oxford prosthesis
BP: Buechel-Pappas
CAD: Computer Aided Drafting
CaFi: Calcaneofibular
CGP: Good Clinical Practice
CT: Computed Tomography
FE: Finite Element
STAR: Scandinavian Total Ankle Replacement
sTiTa: Superior Tibiotalar
TAA: Total Ankle Arthroplasty
TiCa: Tibiocalcaneal
TiNa: Tibionavicular
UHMWPE: Ultra High Molecular Weight Polyethylene
ZTMTA: Zimmer Trabecular Metal Total Ankle
